# Aberrant Expression and Distribution of Enzymes of the Urea Cycle and Other Ammonia Metabolizing Pathways in Dogs with Congenital Portosystemic Shunts

**DOI:** 10.1371/journal.pone.0100077

**Published:** 2014-06-19

**Authors:** Giora van Straten, Frank G. van Steenbeek, Guy C. M. Grinwis, Robert P. Favier, Anne Kummeling, Ingrid H. van Gils, Hille Fieten, Marian J. A. Groot Koerkamp, Frank C. P. Holstege, Jan Rothuizen, Bart Spee

**Affiliations:** 1 Department of Clinical Sciences of Companion Animals, Faculty of Veterinary Medicine, Utrecht University, Utrecht, the Netherlands; 2 Department of Pathobiology, Faculty of Veterinary Medicine, Utrecht University, Utrecht, the Netherlands; 3 Molecular Cancer Research, University Medical Centre Utrecht, Utrecht, the Netherlands; National Institute of Agronomic Research, France

## Abstract

The detoxification of ammonia occurs mainly through conversion of ammonia to urea in the liver via the urea cycle and glutamine synthesis. Congenital portosystemic shunts (CPSS) in dogs cause hyperammonemia eventually leading to hepatic encephalopathy. In this study, the gene expression of urea cycle enzymes (carbamoylphosphate synthetase (CPS1), ornithine carbamoyltransferase (OTC), argininosuccinate synthetase (ASS1), argininosuccinate lyase (ASL), and arginase (ARG1)), N-acetylglutamate synthase (NAGS), Glutamate dehydrogenase (GLUD1), and glutamate-ammonia ligase (GLUL) was evaluated in dogs with CPSS before and after surgical closure of the shunt. Additionally, immunohistochemistry was performed on urea cycle enzymes and GLUL on liver samples of healthy dogs and dogs with CPSS to investigate a possible zonal distribution of these enzymes within the liver lobule and to investigate possible differences in distribution in dogs with CPSS compared to healthy dogs. Furthermore, the effect of increasing ammonia concentrations on the expression of the urea cycle enzymes was investigated in primary hepatocytes in vitro. Gene-expression of CPS1, OTC, ASL, GLUD1 and NAGS was down regulated in dogs with CPSS and did not normalize after surgical closure of the shunt. In all dogs GLUL distribution was localized pericentrally. CPS1, OTC and ASS1 were localized periportally in healthy dogs, whereas in CPSS dogs, these enzymes lacked a clear zonal distribution. In primary hepatocytes higher ammonia concentrations induced mRNA levels of CPS1. We hypothesize that the reduction in expression of urea cycle enzymes, NAGS and GLUD1 as well as the alterations in zonal distribution in dogs with CPSS may be caused by a developmental arrest of these enzymes during the embryonic or early postnatal phase.

## Introduction

**Figure 1:**
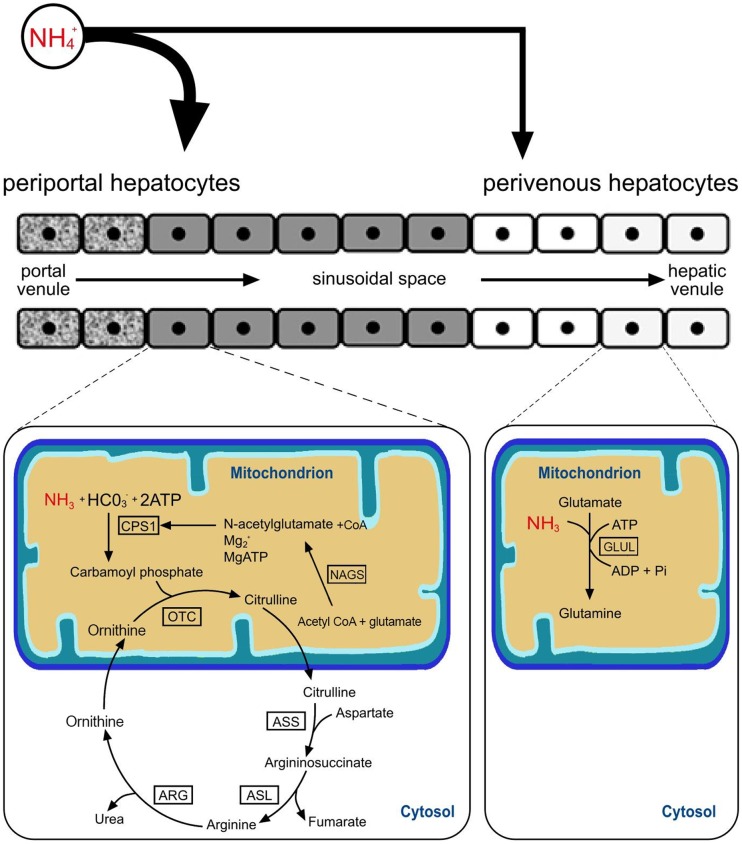
The two major pathways metabolizing ammonia: the urea cycle (periportal), and glutamine synthesis (pericentral). In the urea cycle: ammonia and bicarbonate form carbamoylphosphate via carbamoyl phosphate synthetase1 (CPS1). This reaction requires N-acetylglutamate (acquired via a reaction catalysed by N-acetylglutamate synthase (NAGS)), Mg^2+^ and MgATP. Carbamoylphosphate combines with ornithine in a reaction catalysed by ornithine carbamoyltransferase (OTC) to form citrulline. Citrulline is transported to the cytosol and combines with aspartate to form argininosuccinate (reaction catalysed by argininosuccinate synthetase (ASS1)). Argininosuccinate is then cleaved by argininosuccinate lyase (ASL) yielding fumarate and arginine. Arginase (ARG1) cleaves arginine, producing urea and ornithine. Urea is excreted as waste and ornithine is transported back to the mitochondria to be used in subsequent cycles of urea synthesis. In the pericentral hepatocytes, ammonia ‘escaping’ the urea cycle is metabolized to glutamine (reaction catalysed by glutamate–ammonia ligase (GLUL).

Hyperammonemia is a major factor in the pathogenesis of hepatic encephalopathy (HE) and symptoms including ataxia, stupor, convulsions, and coma have been described in hyperammonemic states. Ammonia detoxification is therefore of utmost importance for maintaining homeostasis, and occurs mainly through two pathways, namely liver-specific urea synthesis (via the urea cycle) and glutamine synthesis. The urea cycle (also known as ornithine or Krebs-Henseleit cycle [1]) is a sequence of biochemical reactions that convert ammonia (NH_3_) into urea ((NH_2_)_2_CO) [2], [3]. The urea cycle is responsible for the disposal of over 90 percent of surplus nitrogen from dietary or endogenous nitrogen sources [4]. It comprises five key enzymes which catalyse the different steps of incorporation of ammonia into urea (Fig. 1). These are carbamoylphosphate synthetase (CPS1; EC 6.3.4.16), ornithine carbamoyltransferase (OTC; EC 2.1.3.3), argininosuccinate synthetase (ASS1; EC 6.3.4.5), argininosuccinate lyase (ASL; EC 4.3.2.1), and arginase (ARG1; EC 3.5.3.1) (Fig. 1). CPS1 and OTC are located on the inner mitochondrial membrane [5], [6] whereas the remaining three enzymes are present in the cytosol [7]. N-acetylglutamate synthase (NAGS; EC 2.3.1.1) is essential for the formation of N-acetylglutamate, an allosteric activator of CPS1, and is therefore indispensable for ureagenesis. Glutamate-ammonia ligase (GLUL; EC 6.3.1.2) represents the other major pathway for ammonia clearance through the production of glutamine from glutamate (Fig. 1) [4]. These two major pathways are distributed in the liver parenchyma in a complementary manner (also known as ‘metabolic zonation’ or ‘functional hepatocyte heterogeneity’) [8]–[11]. In man, rat, and mice the urea cycle enzymes (UCE) are expressed abundantly in periportal hepatocytes, whereas GLUL is present in the pericentral region of the liver lobules (Fig. 1) [7], [12]–[14]. Glutamate dehydrogenase (GLUD1; EC 1.4.1.3) has a central role in nitrogen metabolism and catalyses the oxidative deamination of glutamate to alpha-ketoglutarate and ammonia.

The abundance of mRNAs of UCE, *Nags* and *Glul* in hepatocytes of mice and rats begins in the second half of the embryonic period and reaches adult levels during the early postnatal period [14]–[21]. *Glud1* is expressed much earlier in hepatocytes and is detected as soon as they differentiate from the embryonic foregut [11], [20], [22]. Previous studies have shown that the Wnt/β-Catenin pathway plays a critical role both in gene expression of UCE and GLUL, and in functional liver zonation.[23]–[26].

Both in human and dogs, hyperammonemia is associated with hepatic insufficiency. Like in humans, chronic portal hypertension with acquired portosystemic collateral circulation, e.g. due to chronic hepatitis or cirrhosis, can cause hyperammonemia in dogs. However, in many dog breeds congenital portosystemic shunts (CPSS) are the most frequent cause of hyperammonemia and HE (for a review on CPSS see van Steenbeek et al. [27]). It is therefore clinically relevant to investigate the function of the ammonia metabolizing pathways in such canine liver diseases. CPSS are inherited single large vascular anomalies that directly connect the portal venous system with the systemic venous circulation bypassing the liver parenchyma [28]. This occurs via an intrahepatic (IHPSS) or an extrahepatic portosystemic shunt (EHPSS). CPSS are reported to occur in 0.18 percent of all dogs [29], but the reported incidence varies between 1–5 percent in breeds like the Irish wolfhound and the Cairn terrier [30]–[32]. A hereditary pattern has been documented in several breeds [30]–[34]. In humans, CPSS are extremely rare [35]–[37].

Although the expression of *Cps1, Otc, Ass1, Asl, Arg1, Nags*, *Glul* and *Glud1* and the heterogeneity of the expression of UCE and *Glul* has been extensively studied and reviewed in the mouse and rat liver, studies about these enzymes in companion animals are scarce. Furthermore, CPSS causes nearly complete shunting of portal blood past the liver. Gut-derived ammonia is, therefore, not directly delivered to the liver via the portal blood, but only after mixing with systemic blood via the hepatic artery. As the portal vein in healthy dogs provides up to 80% of the blood flow to the liver (20% is delivered through the hepatic artery [38]), it is not unlikely that the altered blood flow in dogs with CPSS could influence the expression and zonal distribution of the two main ammonia eliminating pathways.

The aim of our study was to investigate the effect of CPSS on the hepatic metabolism of ammonia. To this aim our approach was threefold: first we evaluated the expression of *CPS1, OTC, ASS1, ASL, ARG1*, *NAGS*, *GLUL* and *GLUD1* in dogs with CPSS before and after surgical closure of the shunt. The second was to investigate the distribution pattern of UCE and GLUL in the liver of healthy dogs and dogs with a CPSS. The third was to study the influence of ammonia on the expression of the urea cycle enzymes in primary hepatocytes *in vitro*. We hypothesized that the ammonium concentration in the periportal hepatocytes is a major factor regulating the expression of UCE in dogs with CPSS.

## Patients and Methods

### Animals

Forty six client-owned referral dogs that were diagnosed with CPSS (32 EHPS and 14 IHPS) at the Utrecht University Clinic for Companion Animals were used in this study. CPSS was suspected upon clinical symptoms and increased basal plasma ammonia concentrations as previously described [30]. The diagnosis and localization of the CPSS (IHPSS or EHPSS) was made by ultrasonography and confirmed during surgery.

Control tissues for gene-expression studies were obtained from eight (two for the microarray and six for the qPCR analysis) healthy, mature dogs. Tissue for the primary hepatocyte culture was obtained from one healthy mature dog. Liver samples were harvested as surplus material directly after euthanasia of these dogs that were used for liver-unrelated research projects. The absence of underlying liver disease in these control tissues was confirmed histologically by a board-certified veterinary pathologist (GG).

### Surgery

In all affected dogs the CPSS was surgically ligated. When complete closure could not be achieved, the CPSS was attenuated to the maximum degree that was tolerated without development of portal hypertension (partial closure) [39]–[41]. Wedge biopsies of the liver were routinely taken during surgery (i.e. intra-operative biopsies). Post-operative recovery was evaluated one month after surgery by measuring 12-hour fasting plasma ammonia concentration and performing a rectal ammonia tolerance test [42]. Doppler ultrasonography was performed to examine the site and patency of the attenuated shunt. Dogs that did not achieve a complete recovery were evaluated again three months after surgery. Complete recovery was defined as resolution of all clinical signs, normal fasting plasma ammonia concentrations, a normal rectal ammonia tolerance test and the absence of ultrasonographic evidence for portosystemic shunting. If recovery was complete, at either one or three months after surgery, liver biopsies (i.e. post-operative biopsy) were taken percutaneously using a 14 G true cut biopsy needle under ultrasonographic guidance [43]. Tissue was divided and (a) snap frozen in liquid nitrogen, (b) fixed in RNAlater (Ambion, Austin, TX, USA) for RNA isolation, and (c) fixed in 10% neutral buffered formalin and embedded in paraffin for histopathological evaluation and immunohistochemistry.

### Microarray Analysis

Generation of DNA microarray gene expression data from these samples has been described previously [44]. The data are available from the public gene expression database GEO, accession number GSE39005. Liver tissue of two healthy dogs, 32 dogs with EHPSS and 14 dogs with IHPSS [44] were used for total RNA isolation using an RNeasy Mini Kit (RNeasy Mini kit, Qiagen, Venlo, The Netherlands). Agilent Canine Gene Expression Microarray V1 containing 42,034 60-mer probes in a 4×44 K layout (Agilent Technologies, Diegem, Belgium) was used to determine genome wide expression on 3 µg of total RNA of each sample by co-hybridizing to the common reference, a pool of total RNA from the two healthy liver samples. Microarray analysis was performed in technical replicate dye-swap for each sample as described previously [44]. Expression data were analysed using ANOVA [45]. In a fixed- effect analysis, sample, array and dye effects were modelled. Correction for multiple testing (Permutation F2-test using 5,000 permutations) was performed and p<0.05 after family wise error correction was considered statistically significant. Genes with statistically significant differential expression in EHPSS versus the healthy liver control were further analysed using MetaCore for pathway analysis (GeneGO, Thomson Reuters, New York, NY). From the the general analysis of the statistically differentially expressed genes we selected for UCE and enzymes involved in ammonia metabolizing pathways (CPS1, OTC, ASS1, ASL, ARG1, NAGS, GLUL and GLUD1).

### Quantitative Real-time PCR

Quantitative PCR (qPCR) was performed on RNA samples of liver tissue from healthy controls (n = 6), intra-operative samples (n = 6) representing a functional shunt, and post-operative samples (n = 6) representing a completely closed shunt obtained from the same dogs. Liver tissue from these six dogs with EHPSS was also used for the microarray analysis. Quantitative PCR was performed on *CPS1*, *OTC*, *ASS1*, *ASL*, *ARG1*, *NAGS*, *GLUL*, and *GLUD1*, as described previously [44]. Due to the small number of fully recovered dogs with an IHPSS, a comparison between intra- and post-operative expression levels of the selected enzymes was only made between EHPSS and the healthy control dogs. Normalization of the qPCR data was performed with four reference genes including glyceraldehyde-3-phosphatedehydrogenase *(GAPDH)*, β-2-microglobulin *(B2M)*, ribosomal protein S5 *(RPS5)*, and ribosomal protein L8 *(RPL8)* [*46*]. Primers for reference genes and genes of interest including their optimum temperature are listed in Table 1. Expression levels were normalized by using the average relative amount of the reference genes. For expression analysis on primary hepatocytes the relative gene expression of each gene product (delta-Cq method) was used as the basis for all mRNA comparisons. Undetectable gene expressions were arbitrarily set to Cq 45 for statistical analysis.

**Table 1:** Primers used for qPCR.

Gene	Ensembl TranscriptID	F/R	sequence	T_m_(°C)	Amplicon Size (bp)
*GAPDH*	ENSCAFT00000037560	F	5′-TGTCCCCACCCCCAATGTATC-3′	58	100
		R	5′-CTCCGATGCCTGCTTCACTACCTT-3′		
*B2M*	ENSCAFT00000038092	F	5′-TCCTCATCCTCCTCGCT-3′	61.2	85
		R	5′-TTCTCTGCTGGGTGTCG-3′		
*HNRPH*	ENSCAFT00000028063	F	5′-CTCACTATGATCCACCACG-3′	61.2	151
		R	5′-TAGCCTCCATAACCTCCAC-3′		
*RPS5*	ENSCAFT00000003710	F	5′-TCACTGGTGAGAACCCCCT-3′	62.5	141
		R	5′-CCTGATTCACACGGCGTAG-3′		
*RPL8*	ENSCAFT00000002627	F	5′-CCATGAATCCTGTGGAGC-3′	55	63
		R	5′-GTAGAGGGTTTGCCGATG-3′		
*ARG1*	ENSCAFT00000000605	F	5′-CAACCTGTGTCTTTCCTCCT-3′	61.9	200
		R	5′-GCCAATTCCCAGTTTATCCAC-3′		
*ASL*	ENSCAFT00000017006	F	5′-CTAGAGGTACAGAAGCGG-3′	58	126
		R	5′-TGCTGTTGAGAGTGATGG-3′		
*ASNS*	ENSCAFT00000003490	F	5′-ATACACCAACTGCTGCTTT-3′	55.8	186
		R	5′-GATTATCTCACCATCCACTTTG-3′		
*ASS1*	ENSCAFT00000031736	F	5′-CCTTTACCACGCTCATTTAGAC-3′	60.1	185
		R	5′-ACTTGCACTTTCCCTTCCAC-3′		
*CPS1*	ENSCAFT00000022222	F	5′-TTATAGCGATGACTACCACCAC-3′	57	101
		R	5′-AGCATTCTTGTATCCACTCCA-3′		
*OTC*	ENSCAFT00000022277	F	5′-TTTGGGTGTGAATGAAAGTCTC-3′	61.4	130
		R	5′-TGATGATTGGGATGGATGCT-3′		
*GLUL*	ENSCAFT00000020795	F	5′-TGTATCTTGTCCCTGCTG-3′	60	183
		R	5′-GTATATTCCTGCTCCATTCCA-3′		
*GLUD1*	ENSCAFT00000025535	F	5′-AATTCCAAGACAGGATATCGGG-3′	62	128
		R	5′-TCAGATCCAAGCCCAGGT-3′		
*NAGS*	ENSCAFT00000022856	F	5′-GTTCCAGACCTGCTACC-3′	62	153
		R	5′-CAGCCCGAGGACTACTA-3′		

CPS1, carbamoyl phosphate synthetase 1; OTC, ornithine carbamoyltransferase; ASS1, argininosuccinate synthetase; ASL, argininosuccinate lyase; ARG1, arginase; NAGS, N-acetylglutamate synthase; GLUL, glutamate–ammonia ligase; GLUD1, glutamate dehydrogenase; GAPDH, Glyceraldehyde-3-phosphatedehdrogenase; B2M, b-2-Microglobulin; RPS5, Ribosomal protein S5; RPL8, ribosomal protein L8.

### Immunohistochemistry

Immunohistochemistry (IHC) was performed for CPS1, OTC, ASS1, ASL, ARG1, and GLUL on liver samples of healthy dogs (n = 6), and randomly selected dogs with IHPSS (n = 6) or EHPSS (n = 6). IHC was performed on liver samples of the same CPSS dogs described for the microarray. IHC was performed as described previously [44]. Antibody specifications including antigen retrieval method are listed in Table 2. IHC staining and lobular localization of the enzymes was evaluated. All immunohistochemically stained sections were assessed by one board certified pathologist (GG).

**Table 2:** Antibody characteristics, manufacturers, dilutions and protocol specifications for immunohistochemistry.

Primary Antibody	Manufacturer	Catalogue no.	Dilution	Diluent	Incubation time	Antigen retrieval
**CPS1**	Sigma-Aldrich	HPA021400	1∶650	ABD	O/N 4°C	TE
**OCT**	Abcam	Ab55914	1∶50	PBS/2%BSA	1 hour RT	Citrate
**ASS1**	Aviva Systems Biology	ARP41366	1∶300	PBS/2%BSA	O/N 4°C	Citrate
**ASL**	Sigma-Aldrich	HPA016646	1∶5,000	ABD	O/N 4°C	Citrate
**ARG1**	Lifespan biosciences	LS-C80751/26526	1∶1,000	PBS/2%BSA	1 hour RT	Citrate
**GLUL**	Abcam	Ab73593	1∶2,000	ABD	45 min RT	Citrate

CPS1, carbamoyl phosphate synthetase 1; OTC, ornithine carbamoyltransferase; ASS1, argininosuccinate synthetase; ASL, argininosuccinate lyase; ARG1, arginase; GLUL, glutamate–ammonia ligase; ABD, antibody diluent (DAKO); PBS, Phosphate-buffered saline; BSA, Bovine serum albumin; TE, Tris-Ethylenediaminetetraacetic acid.

### Primary Hepatocyte Culture and Ammonium Chloride Treatment

Isolation of the hepatocytes from liver was performed as described previously [47]. Isolated primary hepatocytes were plated in 24 wells Primaria dishes in a concentration of 1.5×10^5^ viable cells/well (BD Bioscience, Alphen a/d Rijn, The Netherlands) in Hepatozyme Serum Free Medium (SFM) with 10% Fetal Calf Serum and standard antibiotics. One day after plating, cells were treated with ammonium chloride (range 0 to 6,000 µM) in Hepatozyme-SFM with standard antibiotics, and RNA was isolated three hours after treatment. Urea production (QuantiChrom Urea Assay Kit, BioAssay Systems, Hayward, USA) was measured to confirm the functionality of the primary hepatocytes and CyQUANT (Invitrogen) measurement was used to confirm that the cells were equal in number between experiments. Urea and CyQUANT measurements were performed on respectively four and three replicates.

### Statistical Analysis

#### Quantitative PCR

All data were analysed using R statistics package 2.14.0. [48]. The Wilcoxon rank sum test with continuity correction was used for comparison of mRNA expression of *CPS1, OTC, ASS1, ASL, ARG1, NAGS, GLUL*, and *GLUD1* in liver tissue of control dogs and intra-operatively collected liver tissue from dogs with an EHPSS. The Wilcoxon signed rank test with continuity correction was used to test differences in expression in paired samples of dogs with EHPSS obtained intra- and post-operatively. P-values<0.05 were considered significant.

#### Primary hepatocytes

Linear regression was used to study the association between expression of *CPS1, OTC, ASS1, ASL*, and *ARG1* in primary hepatocytes with ammonia concentration (0, 6, 60, 600 or 6,000 µM). Ammonia levels were entered as a factor. Expression levels were log transformed. A stepwise backward method was used to determine the model of best fit based on Akaike’s information criterion. The validity of the final model was checked by studying the residuals on normality and constancy of variance.

### Ethics of Experimentation

All procedures were approved by and performed according to the standards of the Ethical Committee of Animal Experimentation of the Utrecht University (permit ID 2007.III.08.110) and a mandatory written consent from patient owners was also obtained.

## Results

### Gene Expression Analysis of Liver Tissue

**Figure 2:**
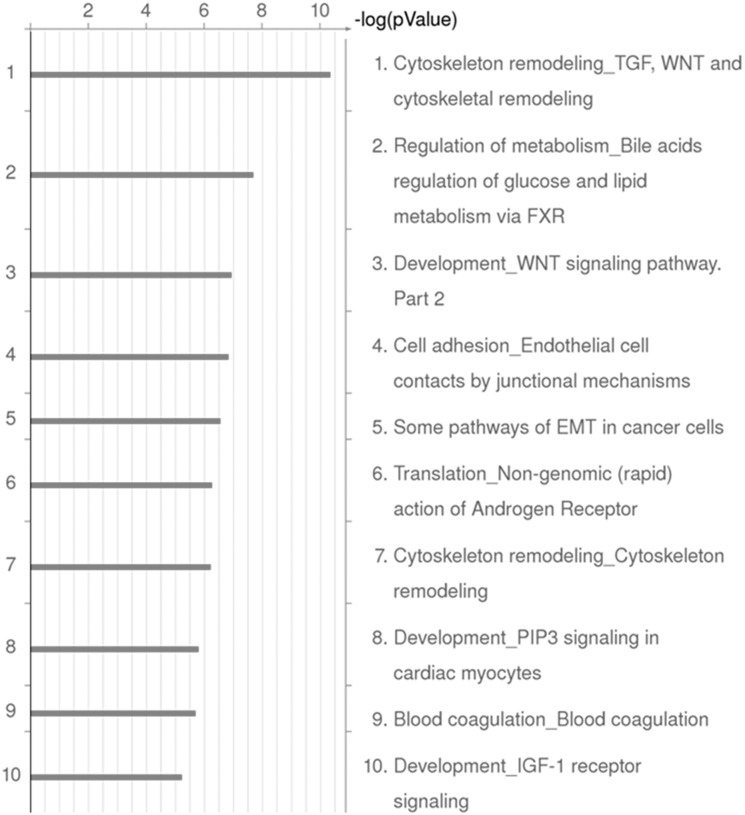
The ten most significantly enriched signalling pathways in dogs with EHPSS. MetaCore pathway analysis based on gene expression differences between healthy dogs and dogs with EHPSS in the microarray analysis.

Pathway analysis of the entire set of genes differentially expressed between CPSS dog and healthy liver showed that the most significant difference in expression of signalling pathways between healthy dogs and dogs with CPSS were related to the Wnt/b catenin pathway (Fig. 2).

The expression of all selected enzymes involved in the ammonia-metabolizing pathways (*CPS1, OTC, ASS1, ASL, ARG1, NAGS, GLUL* and *GLUD1)* was significantly down-regulated in dogs with CPSS compared to healthy liver tissue (Table 3).

**Table 3:** mRNA expression differences of ammonia metabolizing enzymes detected in the microarray.

	EHPSS		IHPSS	
Gene	p-value	M	p-value	M
***CPS1***	<0.01	–0.64	0.0032	–0.40
***OCT***	<0.01	–0.86	<0.01	–0.63
***ASS1***	0.03	–0.23	<0.01	–0.67
***ASL***	<0.01	–0.47	<0.01	–0.60
***ARG1***	<0.01	–0.85	<0.01	–0.66
***NAGS***	<0.01	–0.66	<0.01	–0.53
***GLUL***	0.012	–0.40	<0.01	–0.68
***GLUD1***	<0.01	–1.22	<0.01	–1.21

M value is the average 2 log ratio of gene expression in patients versus healthy control with p-values after multiple testing correction (Methods).

**Figure 3:**
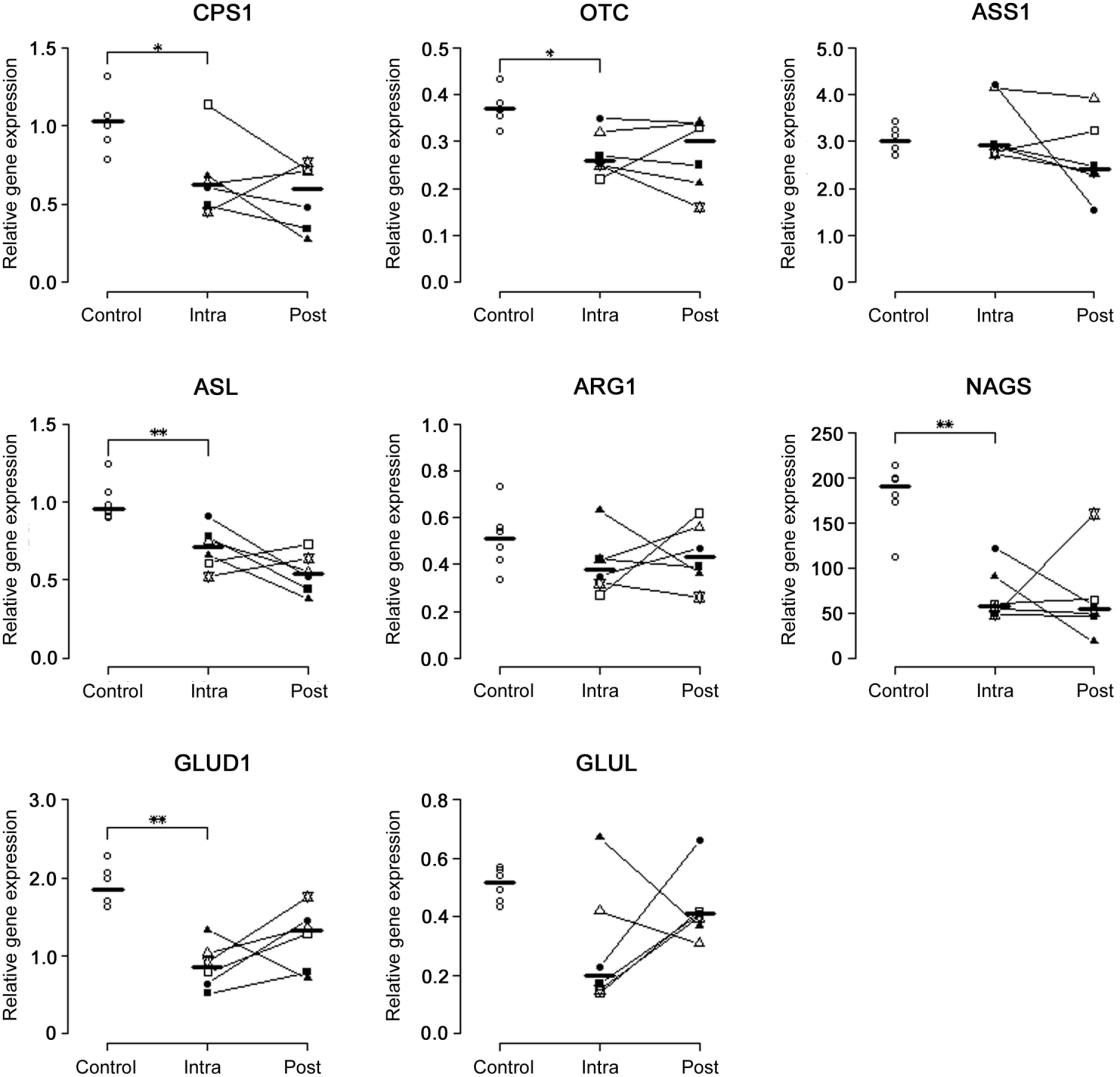
Relative mRNA expression of ammonia metabolizing enzymes in liver tissue of control and CPSS dogs. Significant differences between mRNA expression of the enzymes in control liver samples compared to samples obtained intra-operatively (intra) are indicated by stars (* = p<0.05, ** = p<0.01). There were no significant differences in relative mRNA expression of the enzymes in the liver samples obtained intra-operatively and two months post-operatively (post) in dogs achieving complete recovery.

Replication of the microarray data by performing qPCR in the intra-operative liver tissues confirmed the expression levels of *CPS1* (p = 0.02), *OTC* (p = 0.01), *ASL* (p = 0.004), *NAGS* (p = 0.004), and *GLUD1* (p = 0.002) to be significantly down-regulated in dogs with EHPSS compared to healthy dogs (Fig. 3). No significant differences, however, were found in expression of *ASS1* (p = 0.8), *ARG1* (p = 0.1), and *GLUL* (p = 0.06). No significant changes were found in expression of *CPS1, OTC, ASS1, ASL, ARG1, NAGS, GLUL* and *GLUD1* in CPSS dogs before surgery and after achieving complete recovery (intra-operative vs. post-operative respectively, Fig. 3).

### Immunohistochemistry: Distribution Pattern of the UCE and GLUL

**Figure 4:**
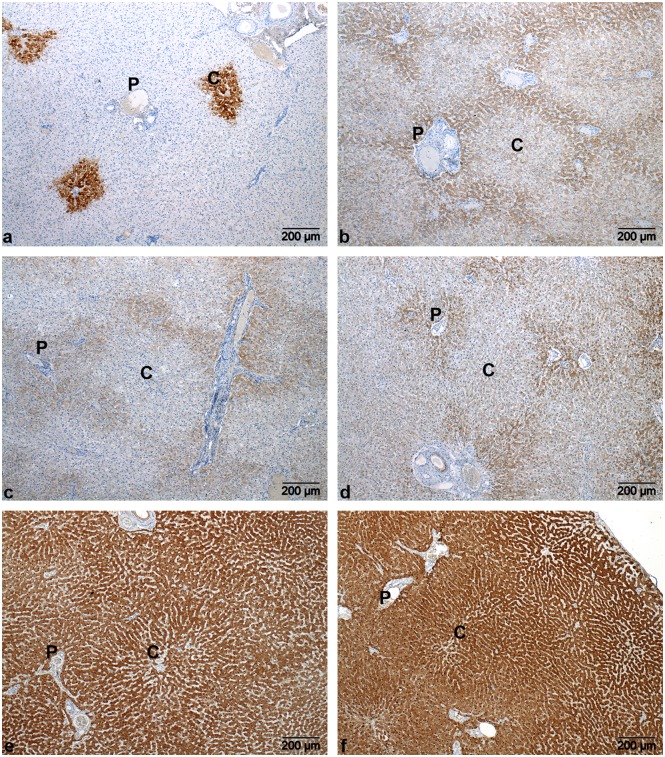
Immunohistochemical localization of ammonia metabolizing enzymes in liver of healthy dogs. GLUL (a) was exclusively expressed immunohistochemically around the central veins (**C**) whereas CPS1 (b), OTC (c) and ASS1 (d) were localized around the portal vessels (**P**). ASL (e) and ARG1 (f) lacked a zonal distribution pattern.

**Figure 5:**
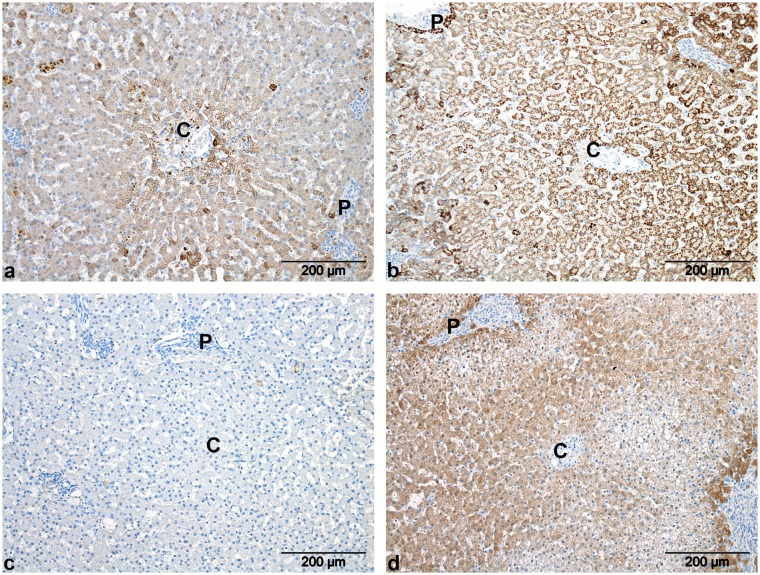
Immunohistochemical localization of CPS1, OTC and ASS1 in liver of dogs with CPSS. In contrast to the periportal distribution of CPS1 in healthy dogs, CPS1 (a+b) showed a slightly more intense staining around the central vein in one dog with an EHPSS (a) and both around the pericentral and periportal vessels in three dogs with an IHPSS (b). No immunoreactivity for OTC was seen in seven dogs with CPSS (c). ASS1 (d) was not distributed according to a zonal pattern as opposed to the periportal distribution seen in the healthy control dogs. In one dog a more intense staining was seen in both periportal and the pericentral areas. C, central vein. P, portal vein.

In all dogs (regardless the phenotype), a clear and consistent pericentral cytoplasmic staining for GLUL was visible in hepatocytes with no staining in other areas of the liver lobule. Typically, the GLUL-immunostained hepatocytes were not concentrically distributed, only a part of the hepatocytes in zone 3 showed immunostaining (Fig. 4a). CPS1 generally showed a more intense staining in the periportal area in the control animals (Fig. 4b). Three dogs with IHPSS also revealed a slightly more intense staining of the pericentral hepatocytes in addition to the periportal staining (Fig. 5b). In three EHPSS dogs, no clear zonal distribution pattern was identified and one animal showed a more prominent pericentral distribution (Fig. 5a). Control dogs generally showed a periportal distribution of cytoplasmic immunoreactivity against OTC (Fig. 4c). However, no OTC immunoreactivity was visible in four animals with EHPSS and in three dogs with IHPSS (Fig. 5c). In the remaining five dogs, 3 dogs had a periportal distribution and in the other two dogs no clear zonal distribution was observed. ASS1 immunoreactivity was generally noted in the cytoplasm of hepatocytes in the periportal area in the control group (Fig. 4d) and the EHPSS group. In the IHPSS group no zonal pattern was detected in three animals. One animal revealed a periportal and pericentral distribution (Fig. 5d) whereas in two animals staining was either evenly distributed in the parenchyma with no distinction between periportal and pericentral areas or the immunoreactivity was patchy as a result of which no zonal pattern could be identified. Tissue sections stained against ASL and ARG1 generally lacked a zonal distribution pattern throughout the liver lobules in all groups (Fig. 4e, f).

### Hepatocyte Culture and Ammonium Chloride Treatment

**Figure 6:**
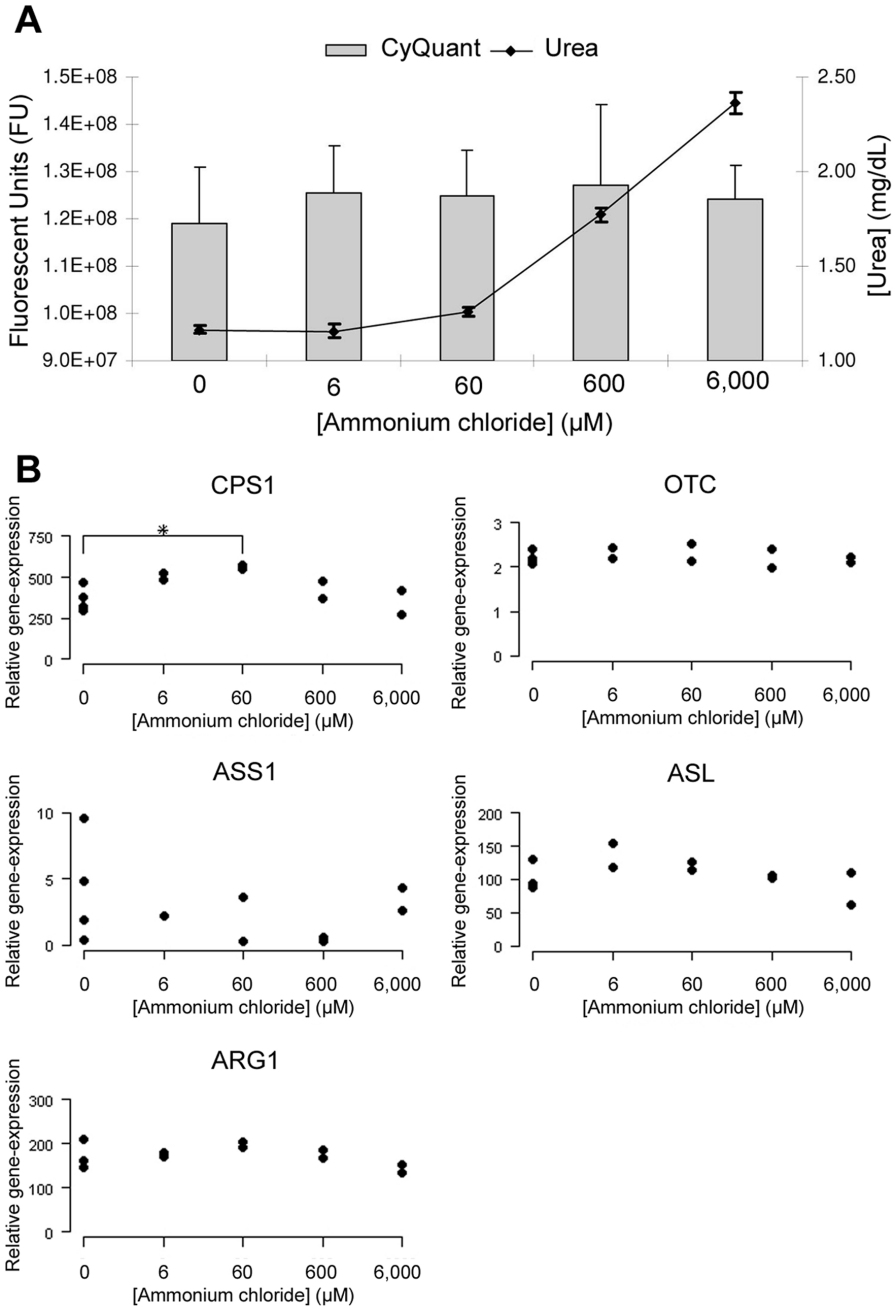
Effect of ammonia on primary hepatocytes. Cell number (n = 3) and urea production (n = 4) in primary hepatocytes after treatment with different ammonia concentrations, data depicts average with standard deviation (**A**). Increased ammonia production and unchanged cell numbers (indicated by CyQUANT) indicate vitality of hepatocytes and active UCE. Relative expression of urea cycle enzymes in primary hepatocytes (control n = 4; treatment n = 2) (**B**). Primary hepatocytes were treated with either 0, 6, 60, 600 or 6,000 µM ammonia for 3 hours.

Increased urea production was observed 24 hours after the addition of ammonium chloride (Fig. 6a) confirming an active urea cycle in the hepatocytes used. No changes in cell number were observed for any of the ammonium chloride concentrations (Fig. 6a). *CPS1* expression was the only gene influenced by ammonia concentration. A concentration of 60 M NH_3_ gave an increase in expression of 57% (95% CI 14–115%), compared to a concentration of 0 M NH_3._ Ammonia concentrations did not significantly influence mRNA expression of *OTC, ASS1, ASL*, and *ARG1* (Fig. 6b).

## Discussion

In this study, mRNA expression of the *CPS1, OTC, ASL, NAGS,* and *GLUD1* was significantly down regulated in the liver of dogs with CPSS compared to healthy dogs. The reduced expression of these genes was identified with microarray analysis and confirmed with qPCR. The qPCR showed no difference in mRNA expression of *ASS1*, *ARG1*, and *GLUL*, whereas these genes showed significant down regulation in the microarray. The use of a common reference pool containing only two control samples in the microarray study and the biological variation in the liver samples might be an explanation for these differences [44]. Therefore additional healthy liver tissue was used for validation in the qPCR experiments. Surprisingly, expression of all down regulated enzymes did not recover to reach more normal values after complete closure of the shunt while plasma ammonium levels did normalize (Table S1). Although not significant, expression of *GLUL* in qPCR showed a clear tendency to be down regulated in the CPSS dogs compared to healthy dogs (p = 0.06) and had a tendency to return to reference levels after closure of the shunt.

One of the mechanisms that we have hypothesized to explain the reduced expression of *CPS1, OTC, ASL, NAGS,* and *GLUD1* was the altered ammonia concentration in the periportal hepatocytes due to the severely reduced portal blood flow (in most cases more than 95% of the portal blood bypasses the liver [49]). However, if mRNA expression of these enzymes is indeed dependent on the ammonia concentration in the periportal hepatocytes, it would be expected that the reduced mRNA expression would increase to normal values once a successful restoration of the portal blood flow has been achieved. In this study, the reduced mRNA expression of *CPS1, OTC, ASL, NAGS,* and *GLUD1* did not return to normal levels after restoration of the portal blood flow. In addition to this, only the mRNA expression of CPS1 in this study increased in response to elevated ammonium levels in primary cultured dog hepatocytes. Therefore, based on these observations, it seems unlikely that an altered ammonia concentration in the periportal hepatocytes is the reason for the reduced enzyme expression in dogs with a CPSS.

Messenger RNA expression of UCE, *NAGS, GLUD1* and *GLUL* is co-ordinately induced during the prenatal period and reaches a steady state (comparable with the adult expression) in the perinatal period [15]–[18]. It seems as if the expression of *CPS1, OTC, ASL, NAGS* and *GLUD1* in dogs with CPSS has come to a developmental arrest which prevented them to normalize after closure of the shunt. Mechanisms that could explain such a phenomenon are mutations in specific transcription factors that would consequently alter (perinatal) transcriptional regulation and lead to reduced enzyme mRNA expressions comparable to what has been published in albino (C^3H^ and C^14CoS^) and juvenile visceral steatosis (JVS) mice [50], [51]. The albino mice lack the postnatal developmental increase of UCE and maintain a reduced (prenatal) level of UCE activity and mRNA expression [52] as well as reduced *Glul* activity [53]. In the JVS mice mRNA levels of UCE were decreased while other liver specific enzymes were normally expressed [54]. In both mice, reduced expression of liver enriched transcription factors (a.o. hepatic nuclear factor 1 (*Hnf-1*), hepatic nuclear factor 4 (*Hnf-4*), CCAAT-enhancer binding protein (*C/Ebp*) [55], and activating protein-1 (*Ap-1*) [56]) were found, and considered to be possibly involved in the reduced expression of UCE at birth. Remarkably, in the same microarray performed for the CPSS dogs, expression of *HNF-*3 (involved in the expression of *CPS1*) [57] and nuclear factor Y (*NF-Y*) (involved in the expression of *ASL* and *NAGS*) [58], [59] was also found to be down regulated (Table S2). It remains to be seen if alterations in these transcription factors also play a role in the reduced enzyme expression in CPSS dogs.

As described for the rat [11], [60], [61], mouse [11] and human [14] liver, the healthy canine liver demonstrates a comparable heterogeneous distribution of UCE and GLUL. The distribution of GLUL was in this respect the clearest and was confined (as in other species) in all dogs to the pericentral zone. The asymmetrical distribution of GLUL around the central vein was, to our knowledge, never reported previously and differs from the distribution pattern in mouse, rat and human liver. This asymmetrical distribution might be caused by differences in microcirculation in the peri-central area between dogs and other mammalian species.

Distribution of CPS1, OTC and ASS1 in healthy dog liver was predominantly observed in the periportal zone, a finding comparable to that reported in livers of rat and mouse. ASL and ARG1 lacked a convincing zonal distribution pattern. This is in contrast to the reported periportal zonal distribution in rat liver, but resembles the human liver where ARG1 was also reported to be homogeneously distributed [62]. A functional implication for these differences in distribution pattern is not clear.

Remarkably, OTC was immunohistochemically undetectable in the majority of dogs with CPSS. Expression of OTC in both the healthy and the CPSS dogs was the lowest among the selected enzymes involved in ammonia metabolism in this study. Furthermore, expression of OTC was also found to be significantly down regulated in CPSS dogs. The absence of immunoreactivity for OTC in the CPSS dogs could therefore be related to a reduction in protein levels of OTC to below the detection limit for IHC. This confirms the reduction in OTC expression levels as indicated by gene-expression profiling.

An intriguing finding is that CPS1 and OTC (in both IHPSS and EHPSS) and ASS1 (IHPSS) appear to lack a clear zonal distribution in CPSS dogs.

A similar distribution is also observed in neonatal rat liver where, in contrast to the adult situation, the pericentral hepatocytes contain both CPS1 and GLUL. The adult type of heterogeneity develops gradually and is clearly seen two days before birth for GLUL and a week after birth for CPS and OTC [11], [20], [60]. Therefore, the absence of a clear heterogeneity in CPSS dogs may resemble the UCE distribution pattern of the neonatal or early postnatal liver.

In a study using transgenic mouse models [25] the Wnt/β-catenin pathway was shown to play a key role in the regulation of liver zonation as CPS1 and GLUL were found to be negative and positive critical targets of β-catenin signalling, respectively. In the same study, blocking the β-catenin signalling resulted in perturbed liver zonation, resulting in a ‘periportal-like’ liver i.e. extension of the expression of the periportal enzymes toward the pericentral area. The expression of CPS1 and OTC in both periportal and pericentral hepatocytes in the CPSS dogs in the present study might therefore be associated with alterations in the Wnt/β-catenin pathway. The reason why these aberrant enzyme distributions are not consistently seen in all CPSS dogs is not clear.

GLUL is the only enzyme that is consistently distributed around the central vein in both healthy and CPSS dogs. As was demonstrated by transplantation studies [63], [64], heterogeneity of GLUL is determined at a very early stage of embryonic life. Consequently, the pericentral compartment of gene expression is usually complete in both mouse and rat around 2 days before birth [11]. Therefore, the unique pericentral distribution of GLUL is not influenced by the present of a shunt, which affects the hepatic portal blood flow only after birth. Although *GLUL* is a positive target gene for the Wnt/β-catenin pathway (in contrast to CPS1), its expression and distribution are probably subject to additional transcriptional mechanisms.

Despite the reduced expression of UCE and NAGS in dogs with CPSS, in which the shunt was closed completely, these dogs exhibited blood ammonia levels within the reference interval already one day after closure of the shunt (Table S1). After closure of the shunt, portal hepatic blood flow normalizes and provides adequate portal blood supply to the liver parenchyma. The maintenance of a normal ammonia concentration shortly thereafter (in spite of persistently reduced expression of ammonia metabolizing enzymes) is most probably explained by the huge reserve capacity of the liver. Even a reduced capacity could then normalize the ammonia concentration, under the prerequisite that portal blood could reach the liver. The trend of GLUL to normalize post-operatively in this study, may suggest that GLUL also contributes to the reduction in ammonia concentration through glutamine formation. Post-operative activation of the enzyme molecules (a process that can be realized in a time range of seconds or minutes [7]) may also explain the post-operative normalization in ammonia levels.

In summary, the present study has shown that dogs with CPSS have not only reduced expressions of UCE (*CPS1, OTC*, and *ASL*), *NAGS* and *GLUD1* but also an aberrant enzyme distribution within the liver lobule. These findings resemble the expression and distribution of the UCE during the neonatal/early postnatal period and might be caused by reduced expression of liver enriched transcription factors. The role of transcription factors (e.g. HNF-3, and NF-Y) or factors involved in the Wnt/β-catenin developmental pathway in the reduced expression of UCE (*CPS1, OTC*, and *ASL*), *NAGS* and *GLUD1* and UCE distribution still remains to be elucidated.

## Supporting Information

Table S1 **Ammonia concentrations before and after surgical closure of the shunt.** Ammonia concentrations in µmol/L, reference intervals 15–45 µmol/L.
(DOCX)

Table S2 **mRNA expression differences of liver enriched transcription factors detected in the microarray.** M value is the average 2 log ratio of gene expression in patients versus healthy control with p-values after multiple testing correction (Methods).
(DOCX)
