# Supplementary material for: Aberrant Expression and Distribution of Enzymes of the Urea Cycle and Other Ammonia Metabolizing Pathways in Dogs with Congenital Portosystemic Shunts
Source: PLoS One. 2014 Jun 19;9(6):e100077. doi: 10.1371/journal.pone.0100077 (PMC4063766; doi:10.1371/journal.pone.0100077)
Supplement: Table S1 — Ammonia concentrations before and after surgical closure of the shunt. Ammonia concentrations in µmol/L, reference intervals 15–45 µmol/L. (DOCX) [file pone.0100077.s001.docx]

| **Dog** | **Breed** | **Gender** | **Ammonia pre-operative** | **Ammonia post-operative** | **Days after operation** |
| --- | --- | --- | --- | --- | --- |
| 1 | Mixed breed | Male | 81 | 12 | 2 |
| 2 | Shih Tzu | Female | >286 | 14 | 8 |
| 3 | Cairn terrier | Female | >286 | <7 | 1 |
| 4 | West highland white terrier | Female | >286 | 11 | 1 |
| 5 | Cairn terrier | Male | 72 | 11 | 2 |
| 6 | Jack russel terrier | Female | >286 | 26 | 2 |

**Table S1. Ammonia concentrations before and after surgical closure of the shunt.**

Ammonia concentrations in µmol/L, reference intervals 15-45 µmol/L.
