# Supplementary material for: Aberrant Expression and Distribution of Enzymes of the Urea Cycle and Other Ammonia Metabolizing Pathways in Dogs with Congenital Portosystemic Shunts
Source: PLoS One. 2014 Jun 19;9(6):e100077. doi: 10.1371/journal.pone.0100077 (PMC4063766; doi:10.1371/journal.pone.0100077)
Supplement: Table S2 — mRNA expression differences of liver enriched transcription factors detected in the microarray. M value is the average 2 log ratio of gene expression in patients versus healthy control with p-values after multiple testing correction (Methods). (DOCX) [file pone.0100077.s002.docx]

|  | **EHPSS** | | **IHPSS** | |
| --- | --- | --- | --- | --- |
| **Gene** | **p-value** | **M** | **p-value** | **M** |
| ***HNF3 beta*** | 0.0002 | -0,18 | <0.0002 | -0,57 |
| ***HNF3 gamma*** | <0.0002 | -0,67 | <0.0002 | -0,60 |
| ***NF-Y alpha*** | <0.0002 | -0,92 | 0,01 | -0,21 |

**Table S2. mRNA expression differences of liver enriched transcription factors detected in the microarray**.

M value is the average 2 log ratio of gene expression in patients versus healthy control with p-values after multiple testing correction (Methods).
